# Physiological Effects of Touching Coated Wood

**DOI:** 10.3390/ijerph14070773

**Published:** 2017-07-13

**Authors:** Harumi Ikei, Chorong Song, Yoshifumi Miyazaki

**Affiliations:** 1 Forestry and Forest Products Research Institute, 1 Matsunosato, Tsukuba, Ibaraki 305-8687, Japan; ikei0224@ffpri.affrc.go.jp; 2 Center for Environment, Health and Field Sciences, Chiba University, 6-2-1 Kashiwa-no-ha, Kashiwa, Chiba 277-0882, Japan; crsong1028@chiba-u.jp

**Keywords:** coated wood, tactile, autonomic nervous activity, prefrontal cortex activity, heart rate variability, heart rate, near-infrared spectroscopy, semantic differential method, physiological relaxation, preventive medical effect

## Abstract

This study examined the physiological effects of touching wood with various coating with the palm of the hand on brain activity and autonomic nervous activity. Participants were 18 female university students (mean age, 21.7 ± 1.6 years). As an indicator of brain activity, oxyhemoglobin concentrations were measured in the left and right prefrontal cortices using near-infrared time-resolved spectroscopy. Heart rate variability (HRV) and heart rate were used as indicators of autonomic nervous activity. The high-frequency (HF) component of HRV, which reflects parasympathetic nervous activity, and the low-frequency (LF)/HF ratio, which reflects sympathetic nervous activity, were measured. Plates of uncoated, oil-finished, vitreous-finished, urethane-finished, and mirror-finished white oak wood were used as tactile stimuli. After sitting at rest with their eyes closed for 60 s, participants touched the stimuli with their palm for 90 s each. The results indicated that tactile stimulation with uncoated wood calmed prefrontal cortex activity (vs. urethane finish and mirror finish), increased parasympathetic nervous activity (vs. vitreous finish, urethane finish, and mirror finish), and decreased heart rate (vs. mirror finish), demonstrating a physiological relaxation effect. Further, tactile stimulation with oil- and vitreous-finished wood calmed left prefrontal cortex activity and decreased heart rate relative to mirror-finished wood.

## 1. Introduction

Humans evolved into their current form over 6–7 million years, beginning with our ancestors’ evolution from a subset of primates [1]. This period represents 99.99% of the span of human evolution, during which time we lived in a natural environment. It is therefore considered that human physiological functions are best adapted to natural environments [2]. The considerable urbanization and artificialization that have occurred since the Industrial Revolution and the rapid spread of modern information technology contribute to the increased “stress state” experienced by humans in modern societies [3]. In 1984, American clinical psychologist Craig Brod coined the term “technostress” [4]. In 2008, it was reported that more than 3.3 billion people—half of the globe’s population—live in an urban environment [5]. In response to these stressful situations, scientific evidence supporting physiological relaxation by exposure to natural stimuli from forests and urban green spaces has accumulated [6]. However, there have been few studies on the relaxation effect derived from familiar, naturally derived stimuli in daily life. Therefore, in the current study, we focused on contact with wood, which has been historically used in indoor environments in Japan.

Wood is a familiar, natural material that has been used in houses and furniture, and it is empirically known to have a relaxation effect on humans. The interest in and expectations of the relaxation effect of wood on humans have increased in recent years, and scientific evidence of this effect is required. In a review outlining the current state of research regarding the physiological effects of wood-derived stimulation on humans [7], it was reported that data on the physiological effects of olfactory stimulation have continued to accumulate, following the development of physiological measurement technology in recent years. The oldest report, published in 1992, focused on the physiological effect of olfactory stimulation with Taiwan cypress oil [8], and several other reports have been published since. In recent years, the physiological relaxation effects of olfactory stimulation by stimuli such as air-dried Japanese cypress wood chips [9], α-pinene [10], and D-limonene [11], which are components derived from wood, have been reported. Several studies on visual stimulation have also been conducted using rooms with different designs and proportions of wood [12,13,14] and large wall panels [15]. 

In contrast, there have been extremely few reports on tactile stimuli. Morikawa et al. [16] reported the differential effects on blood pressure when touching plates of Japanese cypress wood vs. artificial material. Sakuragawa et al. [17] examined differences in the effects of tactile stimulation on human physiology that resulted from materials at different temperatures (cool, room temperature, and warm). They found the following: (1) touching an aluminum plate increased blood pressure, but the increase was inhibited when the aluminum was warmed; (2) touching an acrylic plastic plate increased blood pressure, and a greater rate of increase was observed when the acrylic was chilled; and (3) blood pressure did not change in response to touching objects made of Japanese cypress, Japanese cedar, or oak, and did not increase even when the oak was chilled. These reports are pioneering studies on the physiological effects of tactile stimulation with wood on humans. However, they have limitations in that they only examined blood pressure, which is an index of autonomic nervous activity, as a measure of the physiological response to stimuli. Therefore, in our previous research, we examined the physiological effects on brain activity and autonomic nervous activity of touching uncoated wood vs. other materials with the palm of the hand [18]. In this previous study, as an indicator of brain activity, oxyhemoglobin (oxy-Hb) concentrations were measured in the left and right prefrontal cortices using near-infrared time-resolved spectroscopy (TRS). Heart rate variability (HRV) was used as an indicator of autonomic nervous activity. The results indicated that touching uncoated white oak wood with the palm calmed prefrontal cortex activity and increased parasympathetic nervous activity more than the other materials (marble, tile, and stainless steel), thereby inducing physiological relaxation [18].

As a next step, it is necessary to clarify the influence of touching wood with various coatings on the physiological response, as much of the wood used in our everyday lives is coated. Several studies have reported on the psychological effects of contact with coated wood. For example, Bhatta et al. [19] investigated various coated wood (pine and oak) surfaces through the lateral motion of active fingertip exploration. The results indicated that natural and smooth-finished wood surfaces were perceived as more comfortable and relaxing than varnished and wax coated materials. In addition, Berger et al. [20] examined the subjective responses of touching interior wooden materials with the palm of the hand and sole of the foot. These results showed that oil-finished wood was evaluated as being subjectively preferable to lacquer-coated material and sheet laminated board material. However, there have been no reports on the effects of tactile (i.e., palm of the hand) stimulation with wood with various coatings on the physiological response. 

This study therefore aimed to clarify the effects of touching wood with different coatings with the palm on left and right prefrontal cortex activity, assessed using TRS, and on autonomic nervous activity, assessed using HRV and heart rate.

## 2. Materials and Methods 

### 2.1. Participants

The study participants were 18 female university students (mean age, 21.7 ± 1.6 years). We excluded smokers, those currently in treatment for disease, and those with menstrual period during the study period. All the participants were informed about the aim and procedures of the experiment before providing written informed consent to participate. This study was performed in accordance with the regulations of the Ethics Committee of the Center for Environment, Health and Field Sciences, Chiba University, Japan (Project Identification Code Number: 5).

### 2.2. Study Protocol

**Figure 1:**
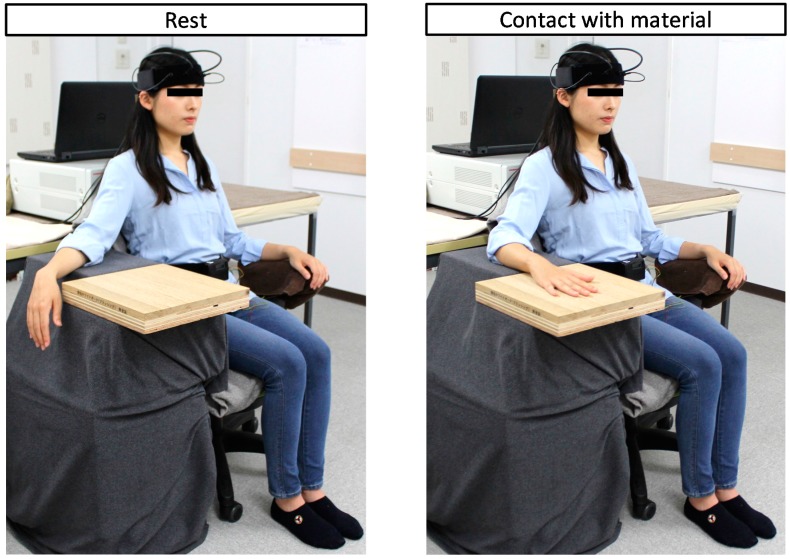
Experimental scene.

**Figure 2:**
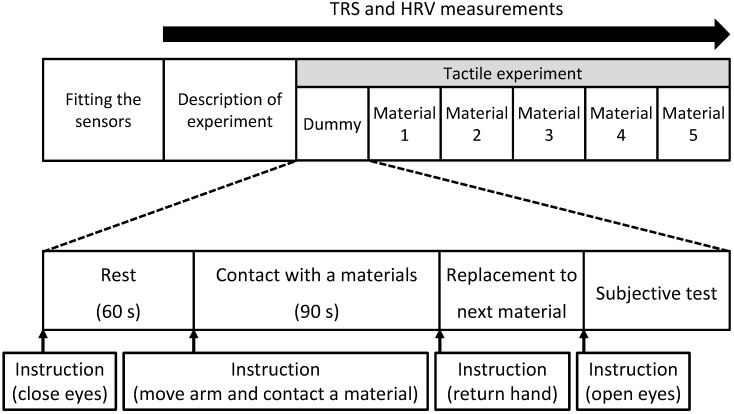
Experimental schedule.

Physiological measurements were performed in a chamber with an artificial climate maintained at 25 °C, 50% relative humidity, and 230-lux illumination. In the waiting room, the participants received a description of the experiment and then moved into the chamber. After sensors for physiological measurement were fitted to the participants’ forehead, they received a description of the measurement procedure while in a seated position. Next, they practiced touching a stimulus with their palm using a dummy sample (sheet laminated flooring). The experimental procedure was as follows. Participants rested with their eyes closed for 60 s (Figure 1, left). Upon receiving instructions from the experimenter, they moved their right forearm using their elbow as a fulcrum and placed the palm on the stimulus (hereafter referred to as “material”) for 90 s (Figure 1, right). After touching the material for 90 s, they returned their hand to the previous position upon the experimenter’s instruction (Figure 1, left). The experimenter then replaced the current material with the subsequent material, hid it with a cloth, and instructed participants to open their eyes. Subsequently, participants completed the subjective evaluation test. Figure 2 shows the experimental schedule. Materials were presented in a counterbalanced order to eliminate any effects due to the order of tactile stimulation. Physiological responses were measured continually.

### 2.3. Tactile Stimulation

White oak (*Quercus alba*) wood was used as the base material. Five laminae without vertical joints (size of each lamina = length 300 × width 60 × thickness 15 mm) were mutually bonded along the width of each wood plate. To prevent bending, a second bonding was performed using Japanese cedar plywood (length 300 × width 300 × thickness 28 mm); the total thickness of the material was 43 mm.

**Figure 3:**
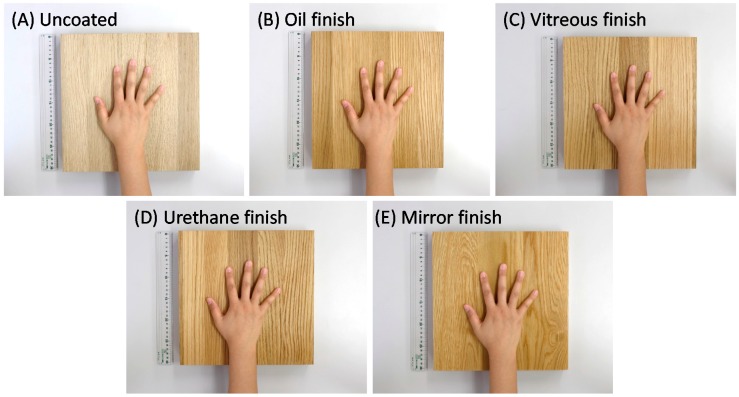
Materials used for the tactile experiment. (**A**) Uncoated wood; (**B**) oil-finished wood; (**C**) vitreous-finished wood; (**D**) urethane-finished wood; (**E**) mirror-finished wood.

The surface of the white oak material was finished by brushing with a stainless-steel wire brush. Additionally, four samples were prepared: (1) uncoated white oak (“uncoated,” Figure 3A); (2) plant oil with an open pore finish extracted from perilla or flax seed applied once as a topcoat to the white oak using a roll coater (“oil finish,” Figure 3B); (3) ordinary temperature glass-coating agent with an open pore finish applied twice as an undercoat and once as a topcoat to the white oak using a brush (“vitreous finish,” Figure 3C); and (4) nitrocellulose lacquer with a semi-open pore finish applied once as an undercoat and two-component polyurethane paint applied once as a topcoat to the white oak using a spray gun (“urethane finish,” Figure 3D). These types of coating were selected as materials representative of those used to coat interior building materials (e.g., floors or walls). Finally, a sample (5) with a strong coating, similar to that used on the surface of a piano, was prepared. The surface of the white oak was sanded with an abrasive-band machine (sandpaper 240 grid; Hitachi Koki Co., Ltd., Tokyo, Japan). This material was coated with nitrocellulose lacquer once as an undercoat using a spray gun. After drying, the surface was polished with an abrasive-band machine (sandpaper 320 grid; Hitachi Koki Co., Ltd., Tokyo, Japan). The above processes (undercoat and polishing treatments) were repeated five times each. Finally, polyurethane resin paint with a closed pore finish was applied once as a topcoat using a spray gun (“mirror finish,” Figure 3E).

All materials were kept at room temperature. The physical properties of the five coating materials are shown in Table 1.

### 2.4. Physiological Measurement

#### 2.4.1. TRS

As an indicator of brain activity, TRS, which is a near-infrared spectroscopy method, was used. Sensors were mounted on the subject’s forehead, and oxy-Hb concentrations in the prefrontal cortex were measured (TRS-20 system; Hamamatsu Photonics K.K., Shizuoka, Japan) [23,24,25]. Oxy-Hb concentrations in the left and right prefrontal cortex were measured before participants touched the materials (pre-measurement) and during the 90 s in which participants touched the materials (post-measurement). Because most data were measured at approximately 1.0–1.2 s, they were transformed by linear interpolation. In addition, all data were calculated as the difference between the averages of the 10-s values before touching.

#### 2.4.2. HRV and Heart Rate

HRV and heart rate were used as indicators of autonomic nervous activity. HRV was analyzed during the periods between consecutive R waves (R–R intervals) on electrocardiograms measured with a portable electrocardiograph (Activtracer AC-301A; GMS, Tokyo, Japan) [26,27]. The power levels of the low-frequency (LF; 0.04–0.15 Hz) and high-frequency (HF; 0.15–0.40 Hz) components of HRV were calculated using the maximum-entropy method (MemCalc/Win; GMS, Tokyo, Japan). HF power reflected parasympathetic nervous activity, and the LF/HF ratio reflected sympathetic nervous activity [28,29]. To normalize HRV parameters across participants, natural logarithmic-transformed values were used in the analysis [30]. The values of ln(HF), ln(LF/HF), and heart rate represented the changes that occurred in each 30 s and the overall mean during the 90 s in which participants touched the samples. In addition, all data were calculated as the difference between the averages of the 30-s pre-measurement values.

### 2.5. Psychological Measurement

The modified semantic differential (SD) method [31] was used to evaluate the psychological effects of touching the five samples. The SD method tests the subjective evaluations of participants through a questionnaire with opposing adjectives, each of which is evaluated on a 13-point scale. Six pairs of adjectives were assessed: “comfortable–uncomfortable,” “natural–artificial,” “relaxed–awakening,” “warm–cold,” “uneven–flat,” and “dry–moist.”

### 2.6. Statistical Analysis

SPSS software version 21.0 (IBM Corp., Armonk, NY, USA) was used for all statistical analyses. Paired *t*-tests with the Holm correction were used to compare physiological responses (1) before vs. after touching each material (i.e., pre- vs. post-measurement) and (2) among the five samples (uncoated, oil finish, vitreous finish, urethane finish, and mirror finish). Wilcoxon signed-rank tests with the Holm correction were applied to analyze the differences in psychological indices (1) before vs. after touching each material (i.e., pre- vs. post-measurement) and (2) among the five samples (uncoated, oil finish, vitreous finish, urethane finish, and mirror finish). In all cases, the significance level was set at *p* < 0.05.

## 3. Results

### 3.1. Physiological Effects

#### 3.1.1. TRS

**Figure 4:**
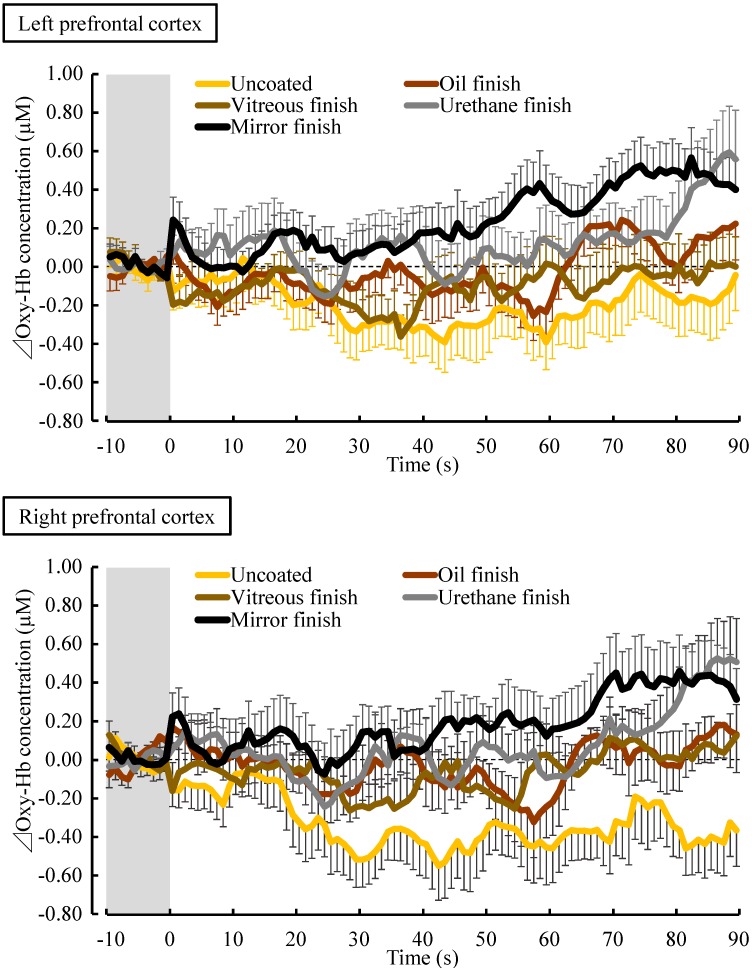
Changes in oxy-Hb concentration in the left and right prefrontal cortices every 1 s over 90 s while touching white oak wood (uncoated, oil finish, vitreous finish, urethane finish, and mirror finish). All data were calculated as the difference between the averages of the 10-s values before touching. Data are expressed as mean ± standard error, *n* = 18.

Figure 4 shows the changes in oxy-Hb concentration per second in the left and right prefrontal cortices while touching the five samples. The mean baseline 10-s oxy-Hb concentration in the left prefrontal cortex did not significantly differ among the five samples before touching the stimuli (uncoated: 43.23 ± 0.95 µM (mean ± standard error); oil finish: 43.38 ± 0.92 µM; vitreous finish: 43.53 ± 0.92 µM; urethane finish: 43.36 ± 0.87 µM; and mirror finish: 42.97 ± 0.89 µM; *p* > 0.05). There was also no significant difference in the baseline 10-s oxy-Hb concentration in the right prefrontal cortex (uncoated: 43.76 ± 1.23 µM; oil finish: 43.13 ± 1.14 µM; vitreous finish: 42.83 ± 1.17 µM; urethane finish: 43.41 ± 1.12 µM; and mirror finish: 43.13 ± 1.13 µM; *p* > 0.05).

Oxy-Hb concentrations in the left and right prefrontal cortices immediately decreased after touching uncoated wood with the palm and remained lower than the pre-measurement value until the end of contact. With mirror finish, oxy-Hb concentrations gradually increased during contact. With oil, vitreous, and urethane finishs, a change was observed between uncoated and mirror finish.

**Figure 5:**
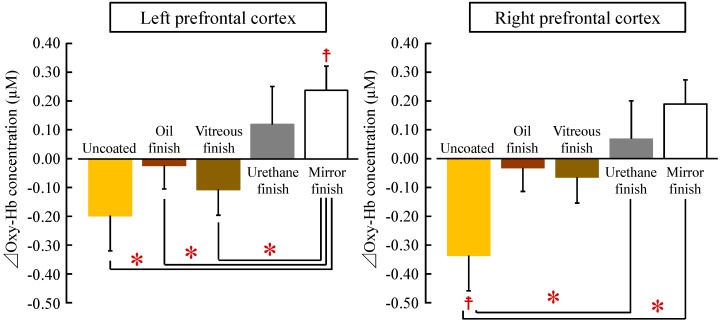
Overall mean oxy-Hb concentrations in the left and right prefrontal cortices while touching white oak wood (uncoated, oil finish, vitreous finish, urethane finish, and mirror finish). Data are expressed as mean ± standard error. *n* = 18, ^☨^ *p* < 0.05 (comparing pre- vs. post-measurement values), * *p* < 0.05 (comparing the five samples) as determined by the paired *t*-test; Holm correction was applied.

Figure 5 shows a comparison of the average differential (post- to pre-measurement) oxy-Hb concentration value in the left and right prefrontal cortices while touching the five samples. Oxy-Hb concentrations in the left prefrontal cortex were as follows: uncoated: −0.20 ± 0.12 µM; oil finish: −0.02 ± 0.08 µM; vitreous finish: −0.11 ± 0.09 µM; urethane finish: 0.12 ± 0.13 µM; and mirror finish: 0.24 ± 0.08 µM (Figure 5, left). Comparing the pre- and post-measurement values, the oxy-Hb concentration after touching wood with mirror finish was significantly increased compared with the pre-measurement value (Figure 5 left, ^☨^ *p* < 0.05). Comparing the five samples, touching uncoated, oil-finished, and vitreous-finished wood significantly decreased oxy-Hb concentrations in the left prefrontal cortex compared with touching mirror-finished wood (Figure 5 left, * *p* < 0.05).

Oxy-Hb concentrations in the right prefrontal cortex were as follows: uncoated: −0.34 ± 0.13 µM; oil finish: −0.03 ± 0.10 µM; vitreous finish: −0.07 ± 0.08 µM; urethane finish: 0.07 ± 0.09 µM; and mirror finish: 0.19 ± 0.13 µM (Figure 5, right). Comparing the pre- and post-measurement values, the oxy-Hb concentration after touching uncoated wood was significantly decreased compared with the pre-measurement value (Figure 5 right, ^☨^ *p* < 0.05). Comparing the five samples, touching uncoated wood significantly decreased oxy-Hb concentrations in the left prefrontal cortex compared with touching urethane-finished and mirror-finished wood (Figure 5 right, * *p* < 0.05).

#### 3.1.2. HRV and Heart Rate

**Figure 6:**
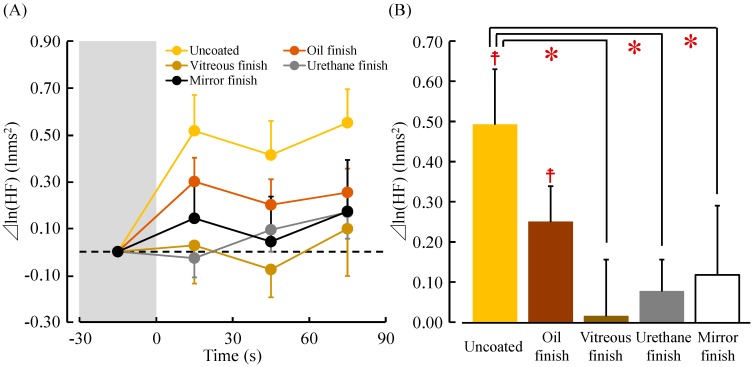
Thirty-second averages and overall mean of the natural logarithm of the HF component of HRV while touching white oak wood (uncoated, oil finish, vitreous finish, urethane finish, and mirror finish). (**A**) Changes in each 30-s average HF value over 90 s; (**B**) Overall mean HF values. Data are expressed as mean ± standard error, *n* = 18, ^☨^ *p* < 0.05 (comparing pre- vs. post-measurement values), * *p* < 0.05 (comparing the five samples) as determined by the paired *t-*test; Holm correction was applied.

Figure 6A shows changes in the ln(HF) value, which reflects parasympathetic nervous activity, while touching the five samples. The mean baseline values of ln(HF) at 30 s before touching the stimuli did not significantly differ among the five samples (uncoated: 5.56 ± 0.23 lnms^2^; oil finish: 5.69 ± 0.21 lnms^2^; vitreous finish: 5.67 ± 0.19 lnms^2^; urethane finish: 5.80 ± 0.19 lnms^2^; and mirror finish: 5.65 ± 0.22 lnms^2^; *p* > 0.05). The ln(HF) value immediately increased after contact with uncoated wood and remained higher than the pre-measurement value or that when touching the four other samples until the end of contact.

Figure 6B shows the average differential (post- to pre-measurement) ln(HF) value while touching the five samples. The ln(HF) values were as follows: uncoated: 0.49 ± 0.14 lnms^2^; oil finish: 0.25 ± 0.09 lnms^2^; vitreous finish: 0.02 ± 0.14 lnms^2^; urethane finish: 0.08 ± 0.08 lnms^2^; and mirror finish: 0.09 ± 0.17 lnms^2^. Comparing the pre- and post-measurement values, the ln(HF) values after touching uncoated and oil-finished wood were significantly increased compared with the pre-measurement values (^☨^ *p* < 0.05). Comparing the five samples, touching uncoated wood significantly increased the ln(HF) value compared with touching vitreous-finished, urethane-finished and mirror-finished wood (* *p* < 0.05).

However, there was no significant difference in ln(LF/HF), which is an index of sympathetic nervous activity, among the five samples (uncoated: −0.69 ± 0.31; oil finish: 0.15 ± 0.23; vitreous finish: −0.05 ± 0.18; urethane finish: −0.40 ± 0.20; and mirror finish: 0.14 ± 0.25; *p* > 0.05).

**Figure 7:**
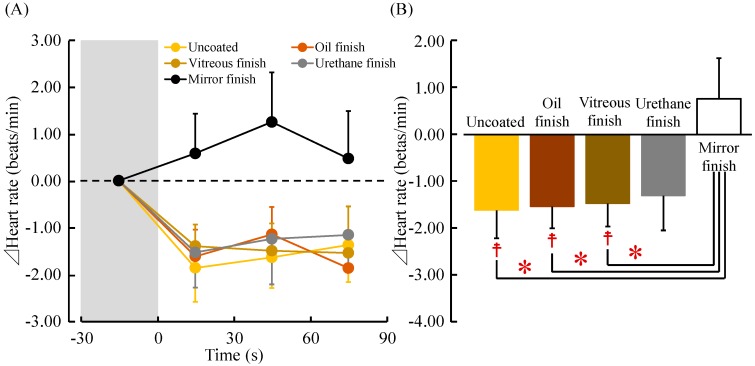
Thirty-second averages and overall mean heart rate while touching white oak wood (uncoated, oil finish, vitreous finish, urethane finish, and mirror finish). (**A**) Changes in each 30-s average heart rate over 90 s; (**B**) Overall mean HF values. Data are expressed as the mean ± standard error, *n* = 18, ^☨^ *p* < 0.05 (comparing pre- vs. post-measurement values), * *p* < 0.05 (comparing the five samples) as determined by the paired *t-*test; Holm correction was applied.

Figure 7A shows the changes in heart rate while participants touched the five samples. The mean baseline heart rate 30 s before touching the stimuli did not significantly differ among the five samples (uncoated: 72.81 ± 2.30 beats/min; oil finish: 72.12 ± 2.28 beats/min; vitreous finish: 73.01 ± 2.05 beats/min; urethane finish: 72.07 ± 2.06 beats/min; and mirror finish: 71.72 ± 2.11 beats/min; *p* > 0.05). Heart rate immediately increased after contact with mirror-finished wood and remained higher than the pre-measurement value and that of the other four samples until the end of contact. However, in uncoated, oil-finished, vitreous-finished, and urethane-finished wood, heart rate decreased after contact and remained lower than the pre-measurement values until the end of contact.

Figure 7B shows the average differential (post- to pre-measurement) heart rate value while touching the five samples. Heart rates were as follows: uncoated: −1.62 ± 0.60 beats/min; oil finish: −1.54 ± 0.46 beats/min; vitreous finish: −1.48 ± 0.49 beats/min; urethane finish: −1.31 ± 0.74 beats/min; and mirror finish: 0.77 ± 0.87 beats/min. Comparing the pre- and post-measurement values, heart rates after touching uncoated, oil-finished and vitreous-finished wood were significantly decreased compared with pre-measurement values (^☨^ *p* < 0.05). Comparing the five samples, touching uncoated, oil-finished and vitreous-finished wood significantly decreased heart rate compared with touching mirror-finished wood (* *p* < 0.05).

### 3.2. Psychological Effects

**Figure 8:**
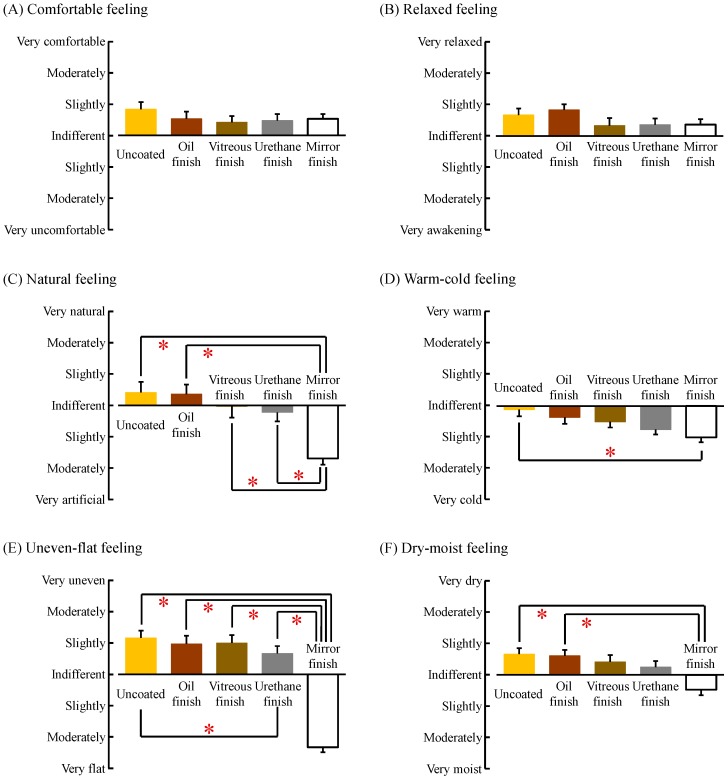
Subjective feelings measured by the modified SD method after touching white oak wood (uncoated, oil finish, vitreous finish, urethane finish, and mirror finish). (**A**) Comfortable feeling; (**B**) relaxed feeling; (**C**) natural feeling; (**D**) warm–cold feeling; (**E**) uneven–flat feeling; (**F**) dry–moist feeling. Data are expressed as mean ± standard error, *n* = 18, * *p* < 0.05 as determined by the Wilcoxon signed-rank test; Holm correction was applied.

The results of subjective evaluation by the modified SD method are shown in Figure 8. Reports of “comfortable” (Figure 8A) and “relaxed” (Figure 8B) feelings did not significantly differ among the five samples (*p* > 0.05). With regard to the “natural” feeling, uncoated and oil-finished wood, which were perceived as “indifferent to slightly natural,” were considered significantly more “natural” than mirror-finished wood, which was perceived as “slightly to moderately artificial” (*p* < 0.05, Figure 8C). In addition, reports of the “natural” feeling were significantly lower after contact with mirror-finished wood compared with vitreous-finished and urethane-finished wood (*p* < 0.05, Figure 8C). With regard to the “warm–cold” feeling, participants reported feeling “indifferent to slightly cold” after contact with uncoated and oil-finished wood, and reported feeling “slightly to moderately cold” after touching mirror-finished wood, representing a significant difference (*p* < 0.05, Figure 8D). With regard to “uneven–flat” feeling, uncoated, oil-finished, and vitreous-finished wood, which were perceived as “slightly uneven,” and urethane-finished wood, which was perceived as “indifferent to slightly uneven,” were considered significantly more uneven than mirror-finished wood, which was perceived as “slightly to moderately flat” (*p* < 0.05, Figure 8E). A significant difference was observed between uncoated and urethane-finished wood on the “uneven–flat” feeling (*p* < 0.05, Figure 8E). Finally, with regard to the “dry–moist” feeling, uncoated and oil-finished wood, which were perceived as “indifferent to slightly dry,” were considered significantly drier than mirror-finished wood, which was perceived as “indifferent to slightly moist” (*p* < 0.05, Figure 8F).

## 4. Discussion

This study aimed to clarify the effects of touching wood with various coatings with the palm on left and right prefrontal cortex activity, assessed using TRS, and on autonomic nervous activity, assessed using HRV. The results were as follows: (1) Tactile stimulation with uncoated wood calmed prefrontal cortex activity (vs. urethane-finished and mirror-finished wood), increased parasympathetic nervous activity (vs. vitreous-finished, urethane-finished, and mirror-finished wood), and decreased heart rate (vs. mirror-finished wood). These results demonstrate a physiological relaxation effect of wood on humans. Further; (2) tactile stimulation with oil- and vitreous-finished wood also calmed left prefrontal cortex activity and decreased heart rate relative to mirror-finished wood; (3) In terms of subjective evaluations, we observed differences among the various coated materials in terms of “natural feeling,” “warm-cold feeling,” “uneven-flat feeling,” and “dry-moist feeling,” and these psychological findings were shown to be consistent with physiological responses. However, for “comfortable” and “relaxed” feelings, which have great value in the subjective evaluation, there was no statistical difference among various types of coated wood. These results clarified the utility of physiologically evaluating the effects of wood on humans.

In this study, touching uncoated, oil-finished, and vitreous-finished wood significantly decreased oxy-Hb concentrations in the left prefrontal cortex compared with touching mirror-finished wood. However, in the right prefrontal cortex, touching uncoated wood significantly decreased oxy-Hb concentrations compared with touching urethane-finished and mirror-finished wood. A difference in left and right prefrontal cortex activity was found. Several previous studies have examined the effect of nature-derived stimuli on prefrontal cortex activity. In these studies, which examined olfactory stimulation with rose, orange [32], and Japanese cypress leaf [33] oils, it was observed that oxy-Hb concentration decreased significantly in the right prefrontal cortex, but there was no significant difference in the left prefrontal cortex. Regarding visual stimulation with real foliage plants [34] and 3D images of the water lily [35], it was also observed that oxy-Hb concentration decreased significantly in the right prefrontal cortex, but there was no change in left prefrontal cortex activity. In contrast, regarding tactile stimulation, it has been shown that touching uncoated Japanese cypress wood significantly decreased oxy-Hb concentrations in the left prefrontal cortex, whereas there was no difference in the right prefrontal cortex [36]. In the current study, there was a significantly greater influence on the left than the right prefrontal cortex; this finding is partially consistent with the results of previous studies on tactile stimulation with wood [36]. These results suggested that tactile stimulation greatly influenced the left prefrontal cortex. However, the underlying mechanism remains unknown. In the future, it will be necessary to accumulate further data and to clarify the difference in left vs. right prefrontal cortex activity.

Regarding the physiological effects of tactile stimulation of wood with the palm, Ikei et al. [18] examined the effects of tactile stimulation with uncoated white oak wood on brain activity and autonomic nervous activity in comparison with other materials (marble, tile and stainless steel). Participants touched the surface of each material for 90 s while sitting with their eyes closed. The results indicated that tactile stimulation with white oak significantly (1) decreased oxy-Hb concentration in the left and right prefrontal cortices relative to marble, tile, and stainless steel and (2) increased ln(HF)-reflected parasympathetic nervous activity relative to marble and stainless steel, thereby inducing physiological relaxation [18]. In addition, Sakuragawa et al. [17] reported differences in the effects of tactile stimulation with several materials, such as wood, on blood pressure. Participants touched the surface of each material for 60 s with their eyes closed. The results indicated the following: (1) Blood pressure increased transiently just after touching oak, Japanese cypress, and Japanese cedar; however, the change did not persist; (2) Blood pressure was high even after the transient increased while touching artificial materials (aluminum or acrylic plastic).

Several reports have been prepared on the physiological effects caused by olfactory stimulation with wood, which are consistent with the findings of our research. Olfactory stimulation with air-dried Japanese cypress wood calmed prefrontal cortex activity compared with high-temperature-dried wood [9]. The effects of olfactory stimulation with Japanese cypress leaf oil on brain activity and autonomic nervous activity have also been investigated [33]. Olfactory stimulation with Japanese cypress leaf oil calmed prefrontal cortex activity and increased parasympathetic nervous activity compared with the control condition (air), indicating that olfactory stimulation with Japanese cypress leaf oil can induce physiological relaxation [33]. Inhalation of α-pinene [10] and D-limonene [11], which are major odor components contained in Japanese cedar and Japanese cypress, increased parasympathetic nervous activity and decreased heart rate compared with control condition (air), indicating physiological relaxation. Our findings on tactile stimulation with uncoated wood are therefore consistent with those of previous studies on tactile and olfactory stimulation by wood or wood-derived materials, demonstrating a physiological relaxation effect of touching natural wood with the palm of the hand.

In the current study, tactile stimulation by uncoated, natural wood and also that by oil- and vitreous-finished wood calmed left prefrontal cortex activity and decreased heart rate relative to mirror-finished wood. This finding can be partially attributed to the differing properties of the coatings tested [37,38]. In terms of physical properties (e.g., surface roughness and heat flow rate), oil- and vitreous-finished wood were similar to uncoated wood. Oil and vitreous finish retain the texture of natural (i.e., uncoated) wood, and it is suggested that this is why tactile stimulation by oil- or vitreous-finished wood induced physiological relaxation.

We believe that tactile stimulation via touching uncoated wood with the palm brought about physiological relaxation effects because physiological functions of modern day humans are best adapted to natural environments or materials. Living in highly urbanized and artificial environments therefore places modern day humans in a state of stress. Accordingly, we enter a relaxed state when exposed to nature-derived stimulation such as wood, which brings us closer to our original natural state. This concept is known as the “back-to-nature” theory [2,39].

In this study, we investigated the effect of contact with coated wood, which is generally used in the built environment, on the human body. Results indicated that touching natural wood or near-natural coated wood with the palm of the hand induced physiological relaxation. We conclude that our finding can serve as a scientific basis for the use of wood, assuming the actual building environment. However, for “comfortable” and “relaxed” feelings, there was no difference among the five samples. This suggests the progress and utility of physiological measurements in biological impact assessment.

In sum, our results revealed a physiological relaxation effect of tactile contact with natural wood (uncoated) or near-natural wood (oil and vitreous finish). However, the study has certain limitations. First, although we clarified the effect of touching wood with the palm of the hand, we did not examine the effect of contact with other body surfaces. Future research should examine the effect of wood on physiological response when touched with the sole of the foot because wood is often used as a flooring material. Second, this study only measured the physiological effects with wood by placing the palm of the hand on the stimulus. It is necessary to clarify the impact of more active forms of contact on the physiological response, such as stroking the surface of the wood with the hand. Finally, the study participants were all female university students in their twenties. Studies on other samples, such as males, children, and the elderly, are therefore required.

## 5. Conclusions

The following conclusions were drawn: (1) Tactile stimulation with uncoated wood calmed prefrontal cortex activity (vs. urethane-finished and mirror-finished wood), increased parasympathetic nervous activity (vs. vitreous-finished, urethane-finished, and mirror-finished wood), and decreased heart rate (vs. mirror-finished wood), demonstrating a physiological relaxation effect of wood on humans; (2) Tactile stimulation with oil- and vitreous-finished wood calmed left prefrontal cortex activity and decreased heart rate relative to mirror-finished wood; However, (3) for the “comfortable” and “relaxed” feelings, there was no difference among the five samples.

## Figures and Tables

**Table 1:** Details of materials. *h*: thickness of materials; *λ*: thermal conductivity; *Ra*: arithmetic average roughness; *JCP*: Japanese cedar plywood.

Coating Type	*h* (mm)	λ (W/(m-K)) ^1^	*Ra* (µm) ^2^	Application Rate (g/m^2^) ^3^
Uncoated	15 (+ JCP 28)	0.120	57.10	–
Oil finish	15 (+ JCP 28)	0.128	56.80	30
Vitreous finish	15 (+ JCP 28)	0.122	57.72	100
Urethane finish	15 (+ JCP 28)	0.119	36.75	644
Mirror finish	15 (+ JCP 28)	0.119	0.12	1400

^1^ A heat flow meter (HFM 436 Lambda; NETZSCH, Selb, Germany), tuned according to ASTM C518-10 [21] and ISO8310 [22], was used. The direction of heat flow was vertically downward. The temperatures of the high- and low-temperature heat plates were 35 °C and 15 °C, respectively. The thermal conductivity at an average material temperature of 25 °C was calculated. Test specimens were used with the cedar plywood attached; ^2^ A contact-surface roughness profilometer (SE3500; Kosaka Laboratory Ltd., Tokyo, Japan) with a diamond needle was used. The evaluation length was 50 mm. The central portion of the samples was measured five times with 50 mm spacing, and the average value was calculated; ^3^ This refers to the attachment mass of the paint per unit area of the surface to be painted.
